# Effect of Vitamin E on Clinical Outcomes in Patients With Non-alcoholic Fatty Liver Disease: A Meta-Analysis

**DOI:** 10.7759/cureus.32764

**Published:** 2022-12-21

**Authors:** Jithin Karedath, Hiba Javed, Fatima Ahsan Talpur, Bihari Lal, Anmol Kumari, Husam Kivan, Venkata Anirudh Chunchu, Shamsha Hirani

**Affiliations:** 1 Internal Medicine, The James Cook University Hospital, Middlesbrough, GBR; 2 Medical School, Dow International Medical College, Karachi, PAK; 3 Medical School, Chandka Medical College, Larkana, PAK; 4 Medicine, Chandka Medical College, Larkana, PAK; 5 Medical School, Ondokuz Mayıs University, Samsun, TUR; 6 Medical School, Avalon University School of Medicine, Willemstad, CUW; 7 Cardiology, Baqai Hospital, Karachi, PAK

**Keywords:** randomized control trials, clinical outcomes, meta-analysis, non-alcoholic fatty liver disease, vitamin e

## Abstract

The aim of the current meta-analysis was to assess the effects of vitamin E on clinical outcomes in individuals with non‐alcoholic fatty liver disease (NAFLD). The current meta-analysis was planned, reported, and conducted per the guidelines of the Preferred Reporting Items for Systematic Reviews and Meta-Analyses (PRISMA) statement. Two authors systematically searched for all papers using PubMed, Cochrane Central Register, and Embase from inception to October 15, 2022. Outcomes assessed in the current meta-analysis included changes in alanine transaminase (ALT) and aspartate transaminase (AST) from baseline in IU/L. Other outcomes included a change in BMI (kg/cm2), a change in total cholesterol level from baseline (mg/l), and a fibrosis score. Total articles were included in the current meta-analysis, enrolling 569 patients (274 patients in the vitamin E group and 295 in the placebo group). The study found that reduction in ALT levels, AST levels, and BMI was significantly greater in patients in the vitamin E group compared to the placebo group. However, no significant differences were reported in terms of change in fibrosis score and total cholesterol.

## Introduction and background

Non‐alcoholic fatty liver disease (NAFLD) is characterized by fatty deposition in the liver cells in individuals without excessive intake of alcohol. It includes a variety of illnesses, from cirrhosis to steatohepatitis to fatty liver [1]. It is one of the most common forms of chronic liver disease worldwide. Since the 1990s, the frequency of NAFLD has more than doubled in adolescents and adults. This chronic liver disease is a substantial cause of morbidity [2]. A meta-analysis conducted by Younossi ZM et al. found that the global prevalence of NAFLD was around 25.24% between 1989 and 2015 [3]. In addition, NAFLD is linked to a significant clinical, financial, and health-related quality of life burden [3].

As the pathogenesis of NAFLD remains poorly understood, NAFLD treatment is far from optimum. The recommended treatment options for NAFLD include weight reduction, regular physical activity, and a healthy diet. Vitamin E is one of the major types of chain-breaking, lipid-soluble antioxidants found in the human body. It occurs naturally in certain foods like seeds, leafy green vegetables, and nuts [4]. Vitamin E is considered a cytoprotective factor. It helps to prevent the liver's degenerative and inflammatory processes during exposure to various dietary factors, environmental pollutants, and xenobiotics [5]. In combination with lifestyle changes, vitamin E may have a potential function in the treatment of NAFLD, according to the literature. However, the limitations of the currently available research prevent us from drawing definitive conclusions on the impact of vitamin E on different clinical outcomes in NAFLD patients [6].

Vitamin E is also known as tocopherol. It is a chain-breaking antioxidant in free radical reactions that is particularly significant in lipid peroxidation and membrane stability. Vitamin E enhances the histology features and biochemistry of NAFLD. A study reported that vitamin E reduced intrahepatic triglycerides through the inhibition of hepatic de novo lipogenesis via its antioxidant activity [7]. Other studies showed that vitamin E lessened NAFLD via multiple other mechanisms, such as protection of cellular structures against upregulation of superoxide dismutase activity, damage from oxygen free radicals, fibrosis, adiponectin, and leptin expression [8-9]. In addition, vitamin E limits membrane injury precipitated by reactive oxygen species (ROS) and is considered a promising antioxidant entity for treating NAFLD [7]. 

One of the most crucial global public health concerns is the rising prevalence of NAFLD. One of the leading causes of the beginning and progression of NAFLD is oxidative stress [10]. Antioxidant therapy has thus been thought to be potentially helpful in treating NAFLD. In particular, vitamin E helps those with NAFLD's histological alterations and liver function [10]. Despite several studies on this topic, there is a dearth of meta-analyses drawing robust conclusions from these studies. Amanullah I et al. carried out a systematic review and reported that vitamin E had beneficial impacts on NAFLD [11]. Since then, certain new studies have been conducted related to the beneficial impacts of vitamin E on NAFLD. In the current meta-analysis, a quantitative synthesis of all these studies has been done to quantify the magnitude of treatment response associated with vitamin E. Current meta-analysis aims to assess the effects of vitamin E in individuals with NAFLD.

## Review

Methodology

The current meta-analysis was planned, reported, and conducted per the guidelines of the Preferred Reporting Items for Systematic Reviews and Meta-Analyses (PRISMA) statement.

Search Strategy

Two authors systematically searched for all papers using PubMed, Cochrane Central Register, and Embase from inception to October 15, 2022. Subject headings were combined with keywords and their synonyms, utilizing search terms like "vitamin E," "Non‐alcoholic fatty liver disease," "clinical outcomes," and "antioxidants." A reference list of all selected articles was also manually searched. Group discussions resolved any discrepancies between the two authors.

Eligibility Criteria

The inclusion criteria were clinical trials assessing the efficiency of vitamin E in patients with NAFLD, irrespective of gender and age, by comparing with placebo or other treatment options. In addition, clinical trials assessing the impact of vitamin E on at least one outcome assessed in the current study were considered for inclusion in the current meta-analysis. Studies were excluded if they were conducted on patients who are pregnant and with co-existing liver illnesses such as hepatitis C, hepatitis B, autoimmune hepatitis, and alcoholic liver disease. We also excluded studies involving drugs like tamoxifen, prednisone, valproate, and amiodarone. Studies other than randomized control trials (RCTs) were also excluded from the current meta-analysis. Studies published in a language other than English were also excluded from the current meta-analysis.

Quality Assessment

The quality of the included RCTs was assessed using the Cochrane Collaboration guidance by two authors independently. The Cochrane Collaboration guidance included the following criteria: random sequence generation, blinding of personnel and participants, allocation concealment, blinding of outcome assessment, incomplete outcome data, selective reporting, and other biases. Group discussions resolved any discrepancies between the two authors.

Outcomes

Outcomes assessed in the current meta-analysis included changes in alanine transaminase (ALT) and aspartate transaminase (AST) from baseline in IU/L. Other outcomes included a change in BMI (kg/cm2), a change in total cholesterol level from baseline (mg/l), and a fibrosis score. The fibrosis score measures the level of scarring to the liver caused by the disease.

Data Extraction

Titles and abstracts were screened to identify relevant studies, followed by full-text screening. Data extraction was done using a pre-defined data extraction form created on Microsoft Excel. Data included in each study included author name, year of publication, sample size, dose, follow-up period, patient characteristics, and outcome data. The second author cross-checked the data and entered it in Review Manager Version 5.4.0 for data analysis.

Data Analysis

Data analysis was done using RevMan version 5.4.0 (The Nordic Cochrane Centre, The Cochrane Collaboration, Copenhagen, Denmark). We estimated the pooled mean differences between the two study groups along with a 95% CI using a random-effect model or fixed-effect model. A p-value <0.05 was considered significant. Heterogeneity among the study results was assessed using I-square statistics. A fixed-effect model was used when I-square statistics was 50% or less, while in the case of I-square less than 50%, a random-effect model was used.

Results

**Figure 1:**
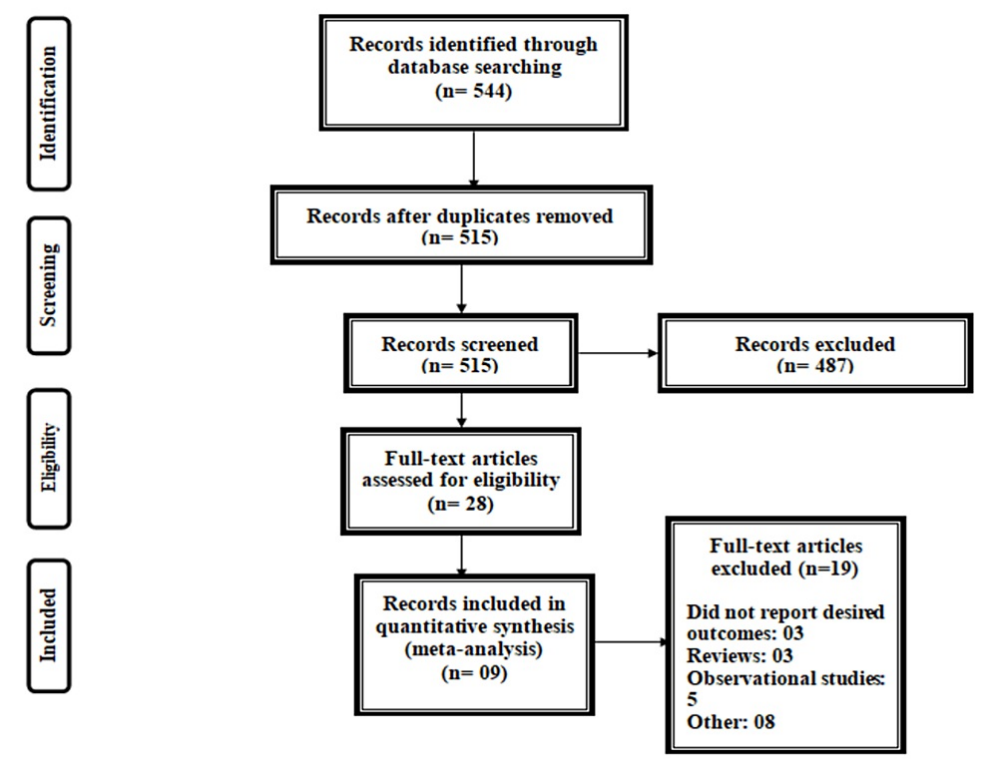
PRISMA flowchart of selection of studies. PRISMA: Preferred Reporting Items for Systematic Reviews and Meta-Analyses.

**Figure 2:**
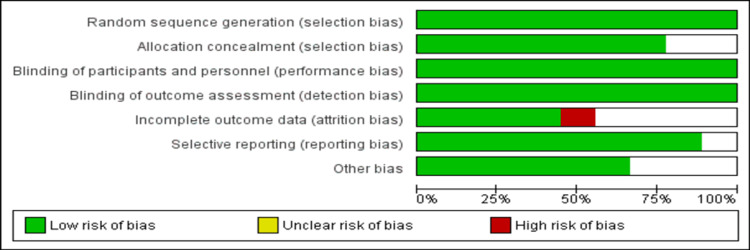
Risk of bias assessment.

Figure 1 summarizes the process of study selection. A total of 544 articles were identified through online searching. After removing duplicates, the title and abstract screening of 515 articles were done. A total of 28 articles were retrieved for full-text screening to assess for eligibility criteria, out of which nine articles were included in the current meta-analysis [12-20], enrolling 569 patients (274 patients in the vitamin E group and 295 patients in the placebo group). Table 1 shows the characteristics of the included studies. The follow-up period of included studies ranged from three months to 30 months. Out of all included studies, four were conducted on adults, and five included children. Figure 2 shows the overall risk of bias assessment of the included studies. The overall risk of bias was low.

**Table 1:** Characteristics of included studies. NR: Not reported.

Author name	Year	Population	Follow-up duration	Groups	Sample size	Mean age (Years)	Males (%)
Aller R et al. [12]	2015	Adults	3 Months	Vitamin E	18	NR	61.10%
				Control	18		
Anushiravani A et al. [13]	2019	Adults	3 Months	Vitamin E	30	NR	NR
				Control	30		
Bril F et al. [14]	2019	Adults	18 Months	Vitamin E	36	58.5	92.64%
				Control	32		
Lavine JE et al. [15]	2011	Children	30 Months	Vitamin E	58	13.25	81%
				Control	57		
Nobili V et al. [16]	2006	Children	12 Months	Vitamin E	43	12.23	31.80%
				Control	45		
Nobili V et al. [17]	2008	Children	24 Months	Vitamin E	25	NR	69.80%
				Control	28		
Pervez MA et al. [18]	2018	Adults	12 Months	Vitamin E	31	44.3	45.31%
				Control	33		
Vajro P et al. [19]	2004	Children	5 Months	Vitamin E	14	10.33	44.50%
				Control	14		
Wang CL et al. [20]	2008	Children	3 Months	Vitamin E	19	13.72	68.40%
				Control	38		

Effect of Vitamin E on ALT

**Figure 3:**
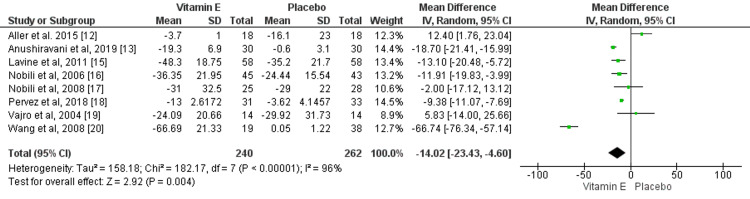
Effect of vitamin E on ALT. Sources: References [12-13, 15-20] ALT: Alanine transaminase.

Eight studies compared the change in ALT levels between patients who received vitamin E and patients who received a placebo. Change in ALT levels was significantly greater in the vitamin E group compared to the placebo group (mean difference: -14.02, 95% CI: -23.43, -4.60), as shown in Figure 3. High heterogeneity was found among the study results (I-square: 96%).

Effect of Vitamin E on AST

**Figure 4:**
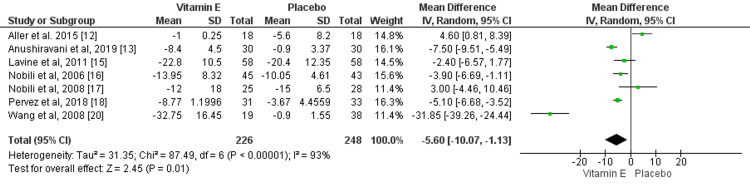
Effect of vitamin E on AST. Sources: References [12-13, 15-18, 20] AST: Aspartate transaminase.

Seven studies compared the change of AST from baseline between patients who received vitamin E and placebo. The mean reduction of AST levels was significantly greater in patients receiving vitamin E compared to placebo (mean difference: -5.60, 95% CI: -10.07, -1.13), as shown in Figure 4. High heterogeneity was found among the study results (I-square: 93%).

Effect of Vitamin E on Fibrosis

**Figure 5:**
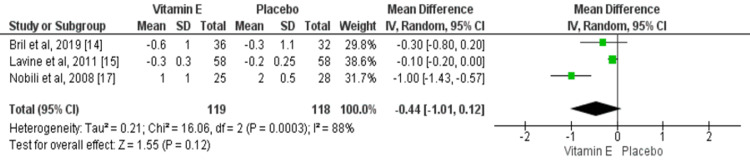
Effect of vitamin E on fibrosis. Sources: References [14-15, 17).

Three studies compared the fibrosis score between two study groups. The pooled mean difference was insignificant between the two study groups (mean difference: -0.44, 95% CI: -1.01, 0.12), as shown in Figure 5. High heterogeneity was found among the study results (I-square: 88%).

Effect of Vitamin E on BMI

**Figure 6:**
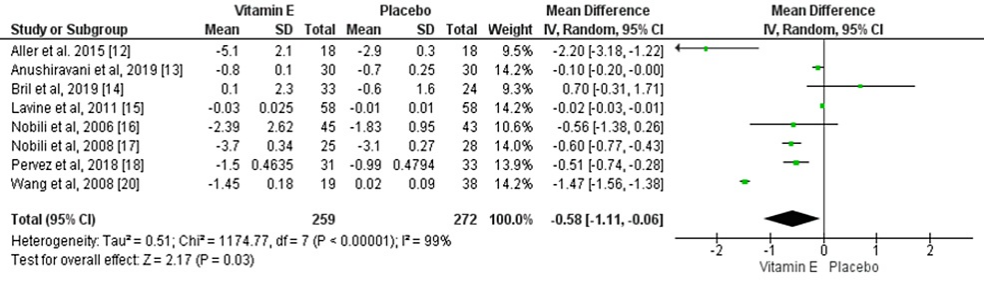
Effect of vitamin E on BMI. Sources: References [12-18, 20].

Eight RCTs assessed the effect of vitamin E on changes in BMI levels. The mean reduction of BMI was significantly greater in patients receiving vitamin E compared to the placebo group (mean difference: -0.58, 95% CI: -1.11, -0.06), as shown in Figure 6. However, high heterogeneity was found among the study results (I-square: 99%).

Effect of Vitamin E on Total Cholesterol

**Figure 7:**
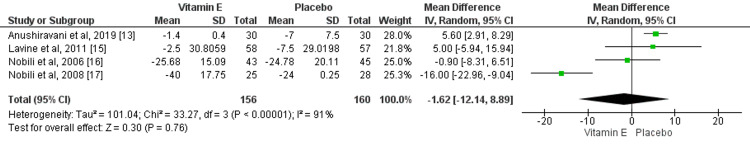
Effect of vitamin E on total cholesterol level. Sources: References [13, 15-17].

Change in total cholesterol level was assessed in four RCTs. No significant difference has been found between the two groups in terms of change in cholesterol from baseline (mean differences: -1.62, 95% CI: -12.14, 8.89), as shown in Figure 7. High heterogeneity was found among the study results (I-square: 91%).

Discussion

Our current meta-analysis aimed to assess the efficacy of vitamin E treatment compared to other treatments in patients affected by NAFLD. The study found that the use of vitamin E in life modification programs adds a significant impact in terms of decreasing ALT levels, AST levels, and BMI. However, no significant differences were reported in terms of total cholesterol and fibrosis score.

NAFLD and metabolic syndrome are closely connected. It may be more severe, and the condition may progress and cause cirrhosis [21]. The core of treatment for individuals with NAFLD includes dietary changes and weight loss [22]. Patients, however, rarely succeed in their lifestyle objectives, and pharmaceutical treatment is typically thought to have the best results [23]. Even though data related to the pathogenesis of NAFLD is rate, oxidative stress can be a major factor in the progression and evolution of NAFLD among patients [6]. With regards to free radical processes like lipid peroxidation, vitamin E is regarded as a chain-breaking antioxidant. It protects against lipid peroxidation by creating complexes with the free radicals' electrons. In this context, vitamin E is regarded as an efficient agent against reactive oxygen species (ROS) and offers defense against liver fibrosis caused by the cytokine "transforming growth factor-beta 1 (TGF-1)" [24].

Among all the included studies that assessed the effect of vitamin E on ALT levels, four studies supported the point that change in ALT is significantly greater in patients receiving vitamin E compared to patients in the control group. Similarly, three RCTs also reported that the change in AST levels is significantly greater in patients receiving vitamin E. Several case-control and cohort studies have been done to assess the impact of vitamin E on NAFLD, but the findings are inconclusive [25]. Erhardt A et al. compared the antioxidant levels of patients with NAFLD with the control. The study concluded reduced levels of tocopherols in NAFLD [6]. A past meta-analysis conducted by Amanullah I et al. showed that the impact of vitamin E is significantly greater in reducing ALT and AST. One of the limitations of this meta-analysis was that the pooled analysis was conducted on just post values of ALT and AST and ignored baseline values [11]. In contrast, the current meta-analysis has focused on changes in ALT and AST values from baseline.

The current meta-analysis did not find a significant difference between patients who received vitamin E and the control group in terms of reducing fibrosis scores. However, only 3 three assessed this outcome and their findings need to be cautiously interpreted as limited by the low sample size.

A study conducted by Hoofnagle JH et al. found that weight loss needs to be the most important priority as obesity can cause worsening of fibrosis [26]. One RCT concluded that vitamin E enhanced ALT and those histological responses were more apparent in individuals with NAFLD who lost weight [26]. The current meta-analysis has also found that the reduction in BMI was significantly greater in patients receiving vitamin E compared to the patients in the control group. The meta-analysis conducted by Amanullah I et al. also found similar results [11].

The current meta-analysis is associated with certain limitations. Firstly, the sample size of the studies was limited. In addition, heterogeneity among the study results in each outcome was high due to variations in vitamin E dosage, formulation, study population, and comparison groups. Despite all these limitations, all included studies were RCTs and covered both children and adults, giving ideas about vitamin E therapy's benefits in both age groups. Considering that the sample size of all studies was very small, it is important that future trials that cover a large population need to be conducted. In addition, future trials should consider the aptness of dosage regimens to develop more recommendations. Secondly, the safety aspect should also be assessed in future studies in children and adults to determine the tolerability of the desired doses of vitamin E.

## Conclusions

The current meta-analysis was conducted to determine the efficiency of vitamin E in enhancing clinical outcomes in patients with NAFLD. The study found that the reduction of ALT, AST, and BMI was significantly greater in patients receiving vitamin E compared to the control group. However, no significant differences were reported between the two groups in relation to fibrosis score and total cholesterol levels. Most included studies were limited, with a low sample size and follow-up duration. However, the current meta-analysis may help to review the guidelines of NAFLD by providing high evidence levels.
